# A99 EFFICACY OF THE IN-HOSPITAL OBSERVATORY PERIOD FOR IBD PATIENTS WITH FLARE TREATED WITH ORAL CORTICOSTEROIDS

**DOI:** 10.1093/jcag/gwac036.099

**Published:** 2023-03-07

**Authors:** J Buttar, S Pi, K Atkinson, B Chou, A Habibi

**Affiliations:** 1 Internal Medicine, University of British Columbia, Vancouver; 2 Gastroenterology, Royal Columbian Hospital, New Westminster; 3 Faculty of Medicine, University of British Columbia, Vancouver, Canada

## Abstract

**Background:**

Inflammatory Bowel Disease (IBD) is a chronic, debilitating collection of diseases with significant impairment to patient quality of life and hospital burden. Patients with acute flare of their disease are managed with IV corticosteroids and ultimately transitioned to an oral equivalent. Despite no formal guideline recommendation, some patients remain in hospital on oral corticosteroids, primarily for observation. Given that each day spent in hospital carries significant costs, examining this observatory period is warranted. The utility of the observatory period for patients recently switched to oral corticosteroids has not yet been studied and may be unnecessarily increasing hospital stays and costs to the Canadian medical system.

**Purpose:**

- Analyse the efficacy of the in-hospital period for patients on oral corticosteroids after an acute IBD flare in preventing recurrence in the 14 and 28 days post discharge.

- Assess the relationship between length of the in-hospital “observatory” period with future hospital burden.

**Method:**

- This retrospective cohort study identified patients with known IBD who were admitted to Royal Columbian Hospital under the Gastroenterology service primarily for acute flare of their disease from June 1, 2020 to June 30^th^, 2022. Patients were identified through the clinic EMR (Plexia) and hospital information was accessed via the Fraser Health Health Records Department. This study acheived ethics approval by the Fraser Health Research Ethics Board (Event: 2022376).

- **Inclusion criteria** are as follows:

- Age > 18 years old

- Prior diagnosis of IBD as per CAG guidelines

- Acute IBD flare is primary reason for hospitalization

- Hospital management includes IV corticosteroids and discharge medications include a tapering course of oral corticosteroid.

- **Exclusion criteria** are as follows:

- Patients found to be non-compliant on outpatient medication regimen (i.e tapering dose of PO prednisone).

- Patients with a complicated course in management (concurrent illness that impacted hospital stay).

**Result(s):**

-20 patients were identified, with 60 total hospital admissions.

-35 visits included an observatory period, with 6 less than 24hr (1 hospital re-presentation), and 29 greater than 24hr (3 hospital re-presentations).

-25 visits did not have an observatory period.

-The relative risk of patients without an observatory period returning to hospital within 14 and 28 days compared to those with an observatory period was 4.2 and 1.75, respectively.

**Conclusion(s):**

These preliminary results suggest an increased relative risk for those without an in-hospital observatory period for short term re-hospitalization. However, we hypothesize this will significantly change once we have broadened our date range and increased our total number of hospitalizations, which is planned prior to the poster presentation date.

**Please acknowledge all funding agencies by checking the applicable boxes below:**

None

**Disclosure of Interest:**

None Declared

